# Effect of Solvent Polarity on the Photo-Induced Polymerization-Induced Self-Assembly of Poly(*tert*-butyl acrylate)-*block*-Polystyrene near Room Temperature

**DOI:** 10.3390/polym18020165

**Published:** 2026-01-07

**Authors:** Tianyi Zhou, Jiawei Song, Gerald Guerin

**Affiliations:** Shanghai Key Laboratory of Advanced Polymeric Materials, Key Laboratory for Ultrafine Materials of Ministry of Education, School of Materials Science and Engineering, East China University of Science and Technology, Shanghai 200237, China

**Keywords:** visible light-initiated, polymerization-induced self-assembly, micelle morphology, solvent polarity

## Abstract

Reversible addition-fragmentation chain transfer mediated polymerization-induced self-assembly (RAFT-PISA) offers an efficient approach for the preparation of polymeric nanomaterials, giving access not only to common structures such as spheres, worm-like micelles and vesicles, but also to much more complex meso-objects. However, when the core forming block polymer possesses a high glass transition temperature (Tg), like poly(methyl methacrylate) or polystyrene (PS), high-order morphologies are particularly difficult to achieve since the glassy core can prevent polymer chain reorganization during PISA. To overcome this issue, we chose to perform visible light-initiated RAFT-PISA of poly(*tert*-butyl acrylate)-*block*-polystyrene (P*t*BA-*b*-PS) in solvent systems with varying degrees of polarity. More specifically, we prepared different mixtures of diisopropyl ether and ethanol and chose P*t*BA as macro-CTA due to its broad range of solubility. By varying the ratio between ethanol and diisopropyl ether, we could observe a transition from spherical micelles to vesicles via intermediate structures (e.g., necklace-like micelles, network-like micellar aggregates and wedding rings). This result was particularly remarkable since the experiments were performed near room temperature. We believe that these multiple morphologies were induced by the interactions between the solvent and the corona and the change in swelling of the polystyrene core with styrene monomer that facilitated its rearrangement. We anticipate that this approach could be applied to other polymeric systems with high Tgs.

## 1. Introduction

When dispersed in a solvent which is good for one block and poor for the other, block copolymers (BCPs) spontaneously arrange themselves in diverse ordered nanostructures (e.g., spheres, lamellae, vesicles) [1]. These nanostructures exhibit broad applications in biomaterials [2], catalysis [3], energy storage materials [4], and beyond. Conventional solution self-assembly techniques for amorphous-amorphous [5,6,7,8] or amorphous-crystalline BCPs [9,10,11] face industrialization challenges due to low solid contents (<1 wt%), multi-step processing, and prolonged preparation time [12]. In contrast, polymerization-induced self-assembly (PISA) permits the one-pot synthesis of block copolymers at high solid contents (10–50 wt%), simultaneously achieving polymerization and morphological diversity without post-processing [13].

PISA is a process in which a polymer dispersed in a good solvent is extended with a second polymer that becomes insoluble as it grows longer, leading to the formation of micelles. As a result, PISA is a dynamic process where the nanostructure morphologies evolve throughout the polymerization of the solvophobic block [14], provided that the insoluble block possesses enough mobility to rearrange as it polymerizes. One of the many advantages of PISA is its versatility since it can be performed via a variety of synthetic methods, such as reversible addition-fragmentation chain transfer (RAFT) polymerization [15,16], atom transfer radical polymerization [17,18], ring-opening polymerization [19,20,21,22], living anionic polymerization [23], etc. Among them, RAFT-mediated PISA (RAFT-PISA) is the most popular [24]. Indeed, RAFT polymerization, which was first mentioned by Rizzardo, Moad, and coworkers [25] in 1998, allows the synthesis of polymers with narrow molecular weight distributions using chain transfer agents to control the polymerization process via a dormancy-activation equilibrium [26].

While RAFT-PISA can be initiated by several approaches, such as thermally [27,28,29,30], by UV [31,32], visible light [33,34] or using enzyme initiation [35,36], most studies have focused on thermally initiated PISA. However, thermally initiated RAFT-PISA often requires high temperature conditions (ca. 70 °C) which are detrimental to the preparation of temperature-sensitive nanomaterials. To overcome this issue, new routes that involve near room temperature initiation have recently been explored [37]. Among these, visible light-mediated RAFT-PISA is particularly appealing due to its mild reaction conditions [38] that enable not only the temporal and spatial control of the reaction via a light “switch” approach [39] but also the use of solvents with low-boiling temperatures. Photo-induced PISA remains, however, extremely challenging to perform at room temperature when the glass transition temperature (Tg) of the insoluble core-forming block is much higher than room temperature, since the rigidity of the micelle core drastically increases as the polymerization processes and its degree of polymerization (DP) becomes larger, strongly limiting the morphological evolution of the final structures to mainly spheres, aggregates of spheres and short worms [40].

Solvent mixtures have already proven to be highly efficient in obtaining various intricate morphologies [7,41,42]. However, this approach has, to date, not been used to unlock new micelle morphologies when the core-forming polymer has a high Tg. To address this gap, we decided to use different solvent mixtures to vary the overall polarity of the polymerization medium and evaluate its impact on photo-initiated PISA. To demonstrate the potential of this approach, we used a challenging and understudied system: poly(*tert*-butyl acrylate)–*block*-polystyrene (P*t*BA-*b*-PS). The reactions were performed near room temperature at a wavelength, λ, of 405 nm. P*t*BA was chosen as the macro-chain transfer (m-CTA) agent because of its broad range of solubility, while PS was used as the core-forming block because it is a well-studied polymer that possesses a high Tg (ca. 100 °C). In addition, the PISA of P*t*BA-*b*-PS block copolymers in alcohol solvent mixtures have not been reported yet. The reactions were performed in different diisopropyl ether/ethanol (DIPE/EtOH) mixtures allowing us to vary the polarity of the solvent system from low (DIPE) to relatively high (EtOH). Although the polymerization was performed near room temperature, far from the Tg of PS, tuning the polarity of the reaction mixture allowed us to fabricate structures with complex morphologies rarely accessible with photo-initiated RAFT-PISA of high Tg polymers.

## 2. Experimental Section

### 2.1. Materials

*Tert*-butyl acrylate (*t*BA, Aladdin, Shanghai, China) was purified by distillation at 55 °C before storage at −30 °C. Chloroform (General-reagent, Shanghai, China, AR), methanol (Adamas-beta, Shanghai, China, AR), diisopropyl ether (General-reagent, AR), ethanol (Adamas-beta, AR), acetone (General-reagent, AR), *n*-hexane (General-reagent, AR), tetrahydrofuran (THF) (General-reagent, AR), NaCl (Titan, Shanghai Titan Technology Co., Ltd., Shanghai, China, 99%) were used as received. Dodecyl 4-(hydroxymethyl) benzyl carbonotrithioate chain transfer agent (CTA) [43] and the photo-initiator sodium phenyl-2,4,6-trimethylbenzoylphosphinate (SPTP) were synthesized as previously reported [44].

### 2.2. Synthesis

We first synthesized a series of P*t*BA macro-CTAs of different lengths by thermal RAFT polymerization (see Supporting Information for a detailed description of the syntheses of these polymers). The average degree of polymerization (DP) and molecular weight distribution index (Ɖ) of the different P*t*BA used as macro-CTAs were determined by nuclear magnetic resonance (NMR) spectroscopy and gel permeation chromatography (GPC). In addition, poly(*tert*-butyl acrylate)-*block*-polystyrene (P*t*BA-*b*-PS) BCPs were synthesized either by thermal RAFT polymerization in or by RAFT-PISA in different solvent mixtures. The Rohrschneider polarity index (P’) was used to evaluate the polarity of the solvent mixtures [45]. Block copolymer micelles were prepared in different DIPE/EtOH mixtures. Thorough description of the polymer syntheses and the calculation of the solvent polarities can be found in Supporting Information.

### 2.3. Experimental Setup

Before starting our experiments, we optimized the reaction conditions. We performed the reactions in a multiwell photoreactor with adjustable power sources that could be varied from 1 to 12 W (PR-6, Shanghai Titan Technology Co., Ltd., Shanghai, China, see Figure S1 in Supporting Information), allowing us to perform several experiments in parallel.

The choice of the reaction temperature was motivated by the fact that the multiwall reactor has its own cooling device that uses the ambient air. Few tests showed that we could obtain a stable temperature throughout the 15 hours of reaction by setting the reaction temperature to 32 °C. SPTP was used as the photo-initiator and the irradiation wavelength was 405 nm. The power source of the photoreactor was set to 5.5 W. Finally, most of the study was performed with P*t*BA_42_ (where 42 indicates the degree of polymerization) as macro-CTA and 25 wt% of solids content.

## 3. Results

As we were particularly interested in evaluating the impact of solvent polarity on the micelle formation by RAFT-PISA, we chose two miscible solvents with significantly different polarity indices, DIPE (P’ = 2.4) and EtOH (P’ = 4.3). We then prepared a series of mixtures by varying the ratio between both solvents to obtain a linear increase in polarity from P’ = 2.4 to 4.3. The impact of polarity of the solvent system on the morphologies of the nanostructures formed was finally evaluated by transmission electron microscopy, and gel permeation chromatography (GPC).

**Figure 1:**
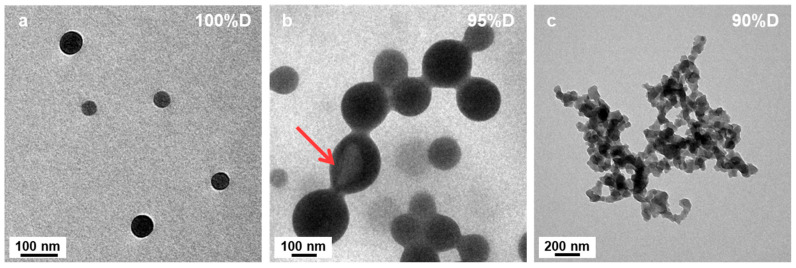
TEM images of P*t*BA_42_-*b*-PS_m_ micelles prepared in binary mixed solvent systems with different volume ratios of DIPE/EtOH. (**a**) 100/0, (**b**) 95/5 and (**c**) 90/10 (*v*/*v*%). The red arrow points at a micelle that looks like a deflated ball. Temperature: 32 °C, solids content: 25 wt%, power: 5.5 W, light source wavelength 405 nm, reaction time: 15 h.

Figure 1 shows TEM images of the resulting P*t*BA_42_-*b*-PS_m_ polymeric micelles after 15 h of irradiation. In pure DIPE (Figure 1a) well-defined spherical micelles of ca. 70 nm diameter could be observed, showing no sign of aggregation. Interestingly, the presence of only 5 v% of EtOH (P’ = 2.49) in the reaction medium during PISA, resulted in micelles that were already significantly different (Figure 1b). While their overall shapes were spherical, these micelles were globally larger (up to 500 nm diameter) and showed a much broader size distribution than those obtained without EtOH. In addition, these micelles were fusing together, forming necklaces, suggesting that the P*t*BA macro-CTA could not fully cover the beads to prevent them from merging [46]. As one should expect, structures of such large sizes were most likely highly swollen, either with monomer or solvent. This could be verified by having a closer look at some of the beads, (see red arrow in Figure 1b, for example) which resembled deflated balls, or opened vesicles.

**Figure 2:**
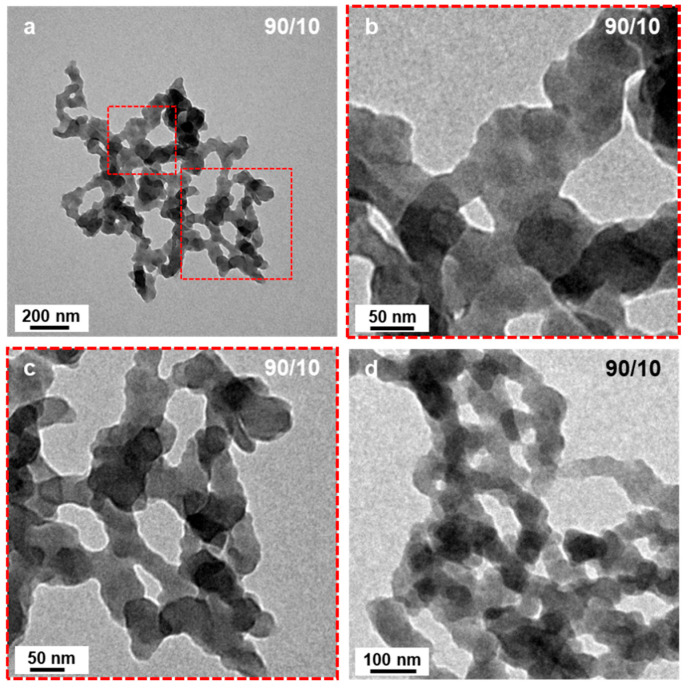
(**a**) TEM image of network-like micellar structures formed by the RAFT-PISA of P*t*BA_42_-*b*-PS_m_ in DIPE/EtOH (90/10, *v*/*v*%). (**b**,**c**) are zoom in TEM images of the sections delineated in (**a**,**d**) is a TEM image of another network. Temperature: 32 °C, solids content: 25 wt%, power: 5.5 W, light source wavelength: 405 nm, reaction time: 15 h.

When the starting solvent mixture contained 10 v% of EtOH (P’ = 2.59), the micelle morphologies changed radically (Figure 1c). No more spheres could be found on the TEM grid, but networks of highly interconnected short worm-like micelles. A closer look at these structures (Figure 2) allowed us to better appreciate their characteristics. We could observe that the ill-defined segments connecting hubs were relatively narrow (ca. 50 nm) and noticeably irregular suggesting that the core was kinetically trapped. Indeed, an increased rugosity would lead to a higher surface free energy induced by the larger interface between the solvent and the micelles, which is thermodynamically unfavorable. It is, however, important to note that, despite being very rare, short, ill-defined worm-like structures had previously been reported. Indeed, György et al. [40] observed such structures when performing thermally induced PISA of poly(lauryl methacrylate)-*block*-poly(methyl methacrylate) (PLMA-*b*-PMMA) experiments in mineral oil or *n*-dodecane with PLMA_22_ used as macro-CTAs and PMMA degrees of polymerizations varying from 69 to 97. The authors attributed this phenomenon to the high glass transition temperature (T_g_) of the PMMA block. These findings are particularly interesting considering that our PISA experiments were performed at relatively low temperature (32 °C) with polystyrene as the solvophobic block which also possesses a high Tg (Tg_∞_ ≈ 100 °C for high molecular weight polystyrene).

**Figure 3:**
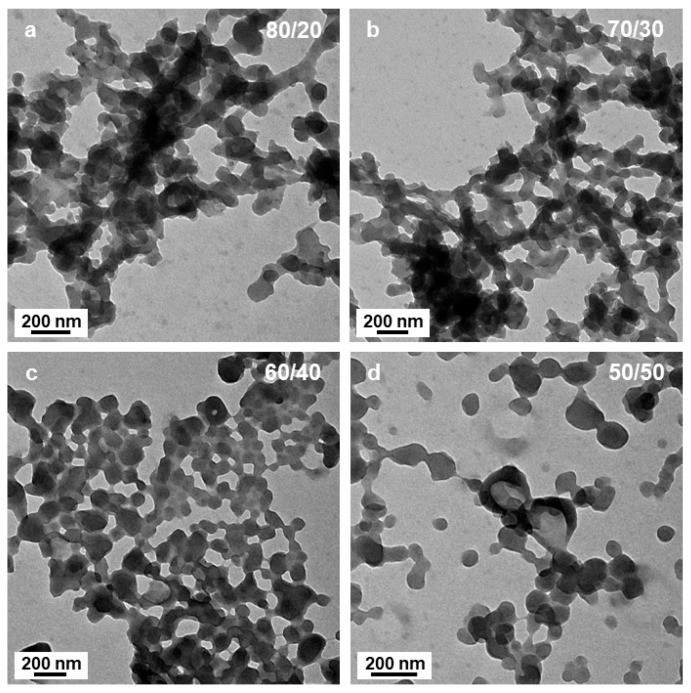
TEM images of P*t*BA_42_-*b*-PS_m_ micelles prepared in DIPE/EtOH with different volume ratios. (**a**) 80/20, (**b**) 70/30, (**c**) 60/40 and (**d**) 50/50 *v*/*v*%. Temperature: 32 °C, solids content: 25 wt%, power: 5.5 W, light source wavelength: 405 nm, reaction time: 15 h.

Decreasing the amount of DIPE from 90 to 70 v% (the polarity index, P’ increasing from 2.59 to 2.97) did not appear to strongly affect the morphology of the samples after 15 h of irradiation (Figure 3a,b), since similar networks with short interconnected ill-defined worm-like segments could be observed. The micelle morphology was more strongly affected by the polarity of the system when P’ increased to 3.16 (DIPE/EtOH 60/40 *v*/*v*%), since we could clearly distinguish the presence of small spheres with diameters close to 20 nm aggregated together. Once again, these structures were extremely similar to those reported by György et al. [40] for PLMA_30_-PMMA_x_ formulations when the DP of the PMMA block reached high values (x = 196, for example).

**Figure 4:**
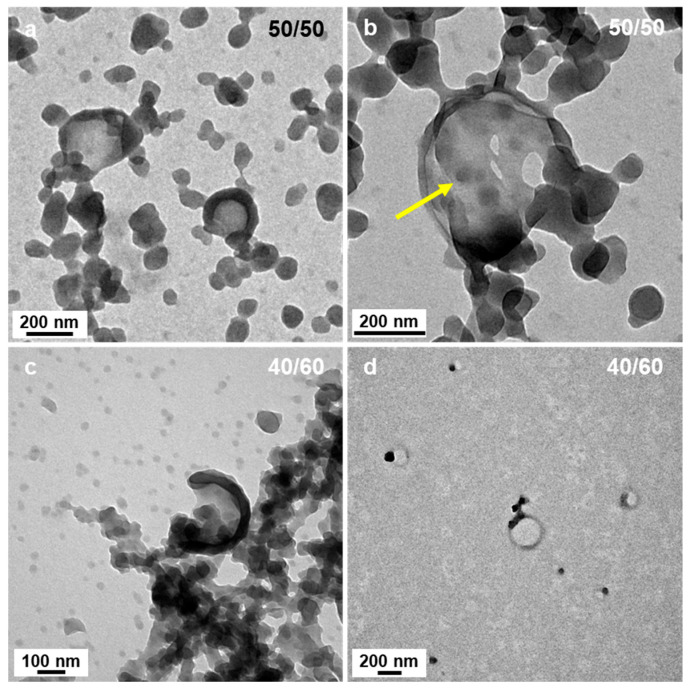
TEM images of P*t*BA_42_-*b*-PS_m_ micelles prepared in DIPE/EtOH with different volume ratios. (**a**,**b**) 50/50 and (**c**,**d**) 40/60 (*v*/*v*%). The yellow arrow points at a small sphere that appears to have partly merged with other spheres to form the flat bottom section of a bowl-like structure. Temperature: 32 °C, solids content: 25 wt%, power: 5.5 W, light source wavelength: 405 nm, reaction time: 15 h.

In most of the cases, however, sphere aggregates represent the final type of nanostructures observed when thermally initiated PISA of high Tg polymers were used. This was not the case here since when the polarity was set to P’ = 3.35 (DIPE/EtOH 50/50 *v*/*v*%), we could also observe some bowl shape nanostructures with a diameter of ca. 200 nm (Figure 4a,b). While the occurrence of these structures was surprising at first sight, a closer look at the aggregated spheres observed when 60% of DIPE was used to prepare the PISA formulation helped us bring some tentative explanations. Indeed, Figure 3c,d shows that in few places, some spheres were forming more or less circular aggregates where the spheres located in the central parts merged and looked like they were somewhat flattened, while those at the periphery appeared to have preserved their overall shapes. One could hypothesize that as these latter spheres merged, they would be thicker than those located in the inner region, leading to bowl shape (or kippah) micelles. This hypothesis was strengthened by the finding of a bowl-like structure that was also containing few spheres at their central parts that were not fully flattened as pointed by the yellow arrow (Figure 4b).

**Figure 5:**
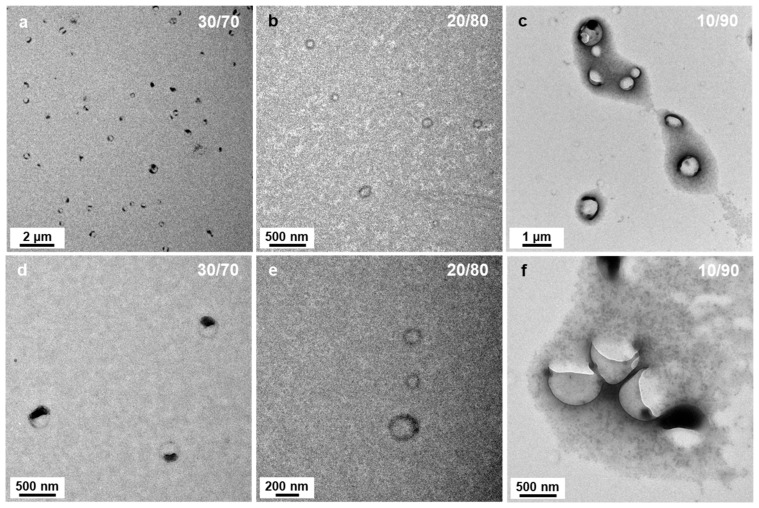
TEM images of P*t*BA_42_-*b*-PS_m_ micelles prepared in DIPE/EtOH with different volume ratios. (**a**,**d**) 30/70, (**b**,**e**) 20/80 and (**c**,**f**) 10/90 (*v*/*v*%). Temperature: 32 °C, solids content: 25 wt%, power: 5.5 W, light source wavelength: 405 nm, reaction time: 15 h.

As the solvent polarity was further increased to P’ = 3.54 (DIPE/EtOH 40/60 *v*/*v*%), not only could we still find aggregated spheres and bowl-like objects, but another type of morphology emerged in the background (see Figure 4d): “wedding rings” forming pale circles bearing one or two nanospheres. As we saw these rings on the TEM, we first believed that they were drying artefacts, and were tempted to ignore them. However, when we used solvent ratios of DIPE/EtOH = 30/70 *v*/*v*% and 20/80 *v*/*v*% (P’ = 3.73 and 3.92, respectively), these wedding rings became prominent and could not be dismissed (Figure 5a,d and Figure 5b,e, respectively). The rings were broadly dispersed in diameter ranging from ca. 50 to 150 nm. When the PISA formulation contained 90 v% EtOH (P’ = 4.11), we mainly found large broken vesicles surrounded by debris (Figure 5c,f).

**Figure 6:**
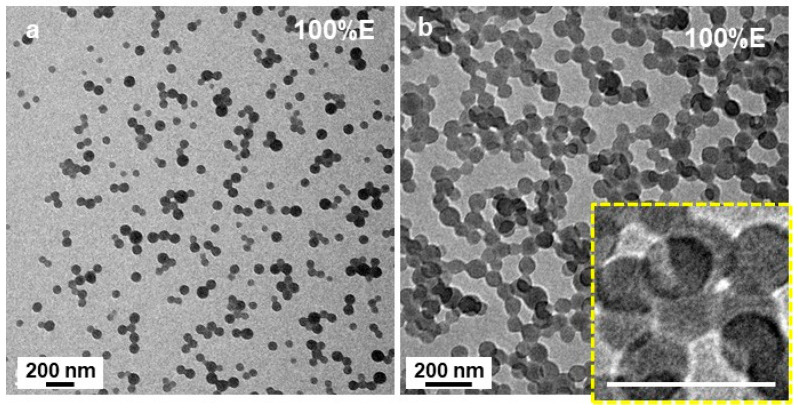
(**a**,**b**) TEM image of P*t*BA_42_-*b*-PS_m_ micelles prepared in pure ethanol. The scale bar in inset of (**b**) is 100 nm. The inset of (**b**) shows a higher magnification of the small beads emphasizing their vesicular characteristics. Temperature: 32 °C, solids content: 25 wt%, power: 5.5 W, light source wavelength: 405 nm, reaction time: 15 h.

Finally, spherical structures were observed when the PISA formulation contained 100 v% of EtOH (P’ = 4.3) (Figure 6). While some well-defined, isolated spheres with diameters close to 40 nm could be observed, (Figure 6a), most of them were incorporated into much larger structures such as, clusters (Figure 6b), networks and even large monoliths. However, a closer look at the some of the clusters (inset of Figure 6b) shows that they were most likely vesicular.

**Figure 7:**
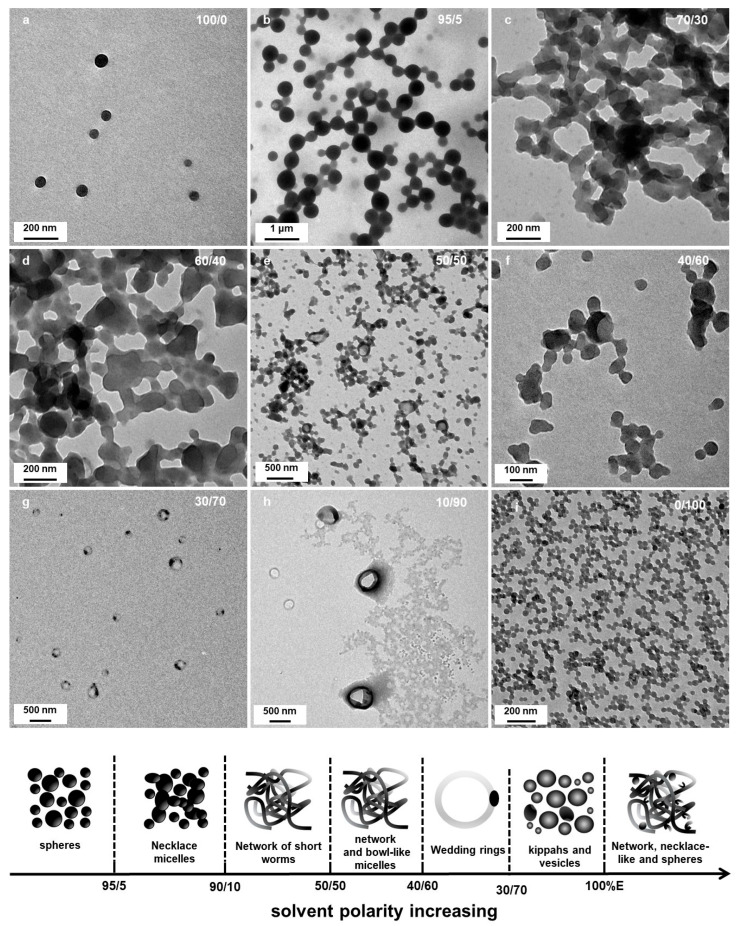
Representative TEM images of P*t*BA_42_-*b*-PS_m_ micellar morphological transitions in different DIPE/EtOH ratios. (**a**) 100/0 (**b**) 95/5; (**c**) 70/30, (**d**) 60/40, (**e**) 50/50, (**f**) 40/60, (**g**) 30/70, (**h**) 10/90 and (**i**) 0/100. Temperature: 32 °C, solids content: 25 wt%, power: 5.5 W, light source wavelength: 405 nm, reaction time: 15 h.

**Figure 8:**
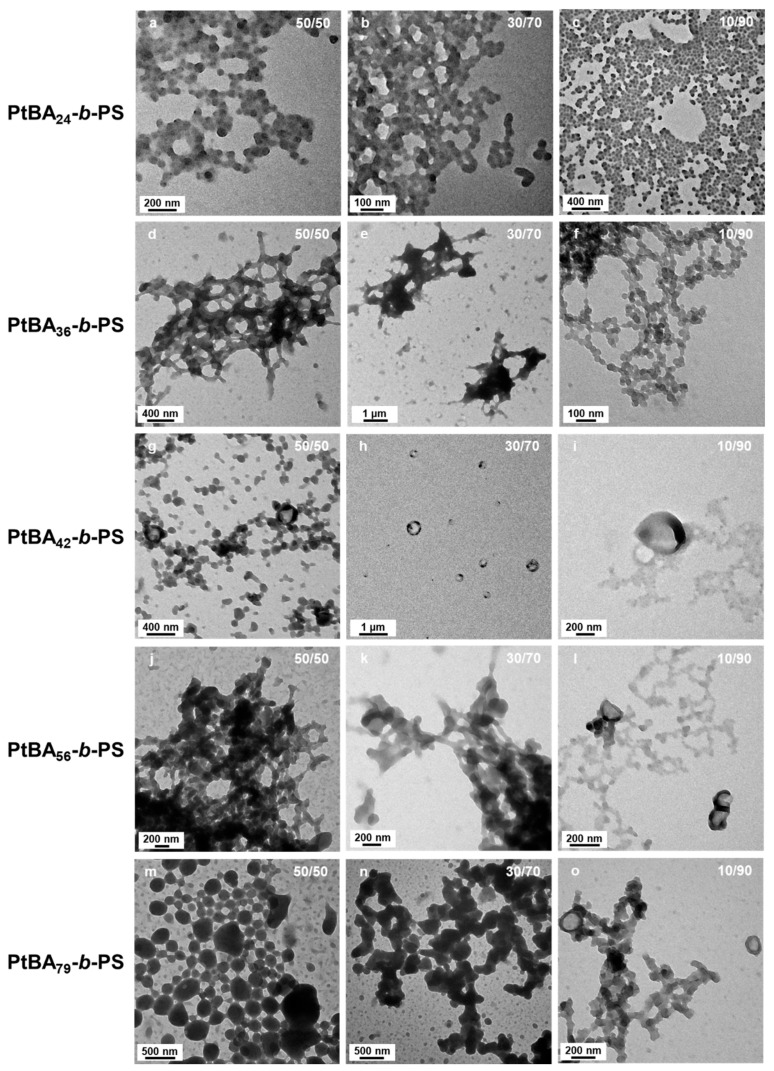
TEM images of P*t*BA-*b*-PS micelle morphology prepared by mP*t*BA with a DP of (**a**–**c**) 24, (**d**–**f**) 36, (**g**–**i**) 42, (**j**–**l**) 56 and (**m**–**o**) 79 in different ratios of mixed solvents: (**a**,**d**,**g**,**j**,**m**) DIPE/EtOH (50/50 *v*/*v*%); (**b**,**e**,**h**,**k**,**n**) DIPE/EtOH (30/70 *v*/*v*%); (**c**,**f**,**i**,**l**,**o**) DIPE/EtOH (10/90 *v*/*v*%). Temperature: 32 °C, solids content: 25 wt%, power: 5.5 W, light source wavelength: 405 nm, reaction time: 15 h.

Building upon our investigation of solvent polarity effects on micellar morphology that are summarized in Figure 7, we further examined how the degree of polymerization of mP*t*BA macro-chain transfer agents (macro-CTAs) influenced the structural characteristics of self-assembled block copolymer micelles in selective solvent systems. A series of mP*t*BA macro-CTAs with varying DPs (24, 36, 42, 56, and 79) were employed. Since we were particularly interested in studying the formation of the less common structures (wedding rings and bowl-shaped micelles), we prepared micelles in solvent systems relatively polar, i.e., containing 50, 30 and 10 v% of DIPE (Figure 8). All the reactions were conducted for 15 h at 32 °C.

Micelles obtained from mP*t*BA_24_ formed aggregates of small merged spheres with no sign of other morphologies, even when the polarity was equal to 4.11.

When the degree of polymerization of mP*t*BA was increased to 36 (Figure 8d–f), the aggregated spheres where no longer visible. Instead, the structures obtained were networks. When the solution contained 50 v% of DIPE, networks of short worms could be seen. As the amount of EtOH was increased to 70 v%, large ill-defined structures, along with pale wedding rings could be found on the TEM grid. Interestingly, when the solvent system was DIPE/EtOH 10/90 *v*/*v*%, the network was made of strings of relatively well-defined spheres.

**Figure 9:**
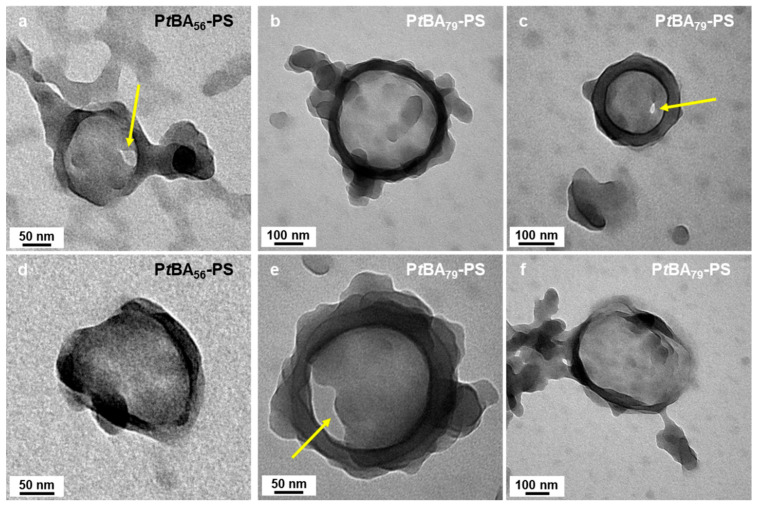
TEM images of bowl-like micelles obtained in DIPE/EtOH 10/90 *v*/*v*% using either (**a**–**c**) mPtBA_56_ or (**d**–**f**) mPtBA_79_ as macromonomer. The yellow arrows point at holes in these structures. Temperature: 32 °C, solids content: 25 wt%, power: 5.5 W, light source wavelength: 405 nm, reaction time: 15 h.

Higher-order architectures, including kippah structures and vesicles, were observed when we used mP*t*BA_42_, mP*t*BA_56_, and mP*t*BA_79_ as macro-CTA (Figure 8g–o). Micelles prepared with mP*t*BA_56_ and mP*t*BA_79_ in DIPE/EtOH (10/90, *v*/*v*%) (Figure 8l,o) exhibited similar structural features to those of mP*t*BA_42_ in DIPE/EtOH (50/50, *v*/*v*%) (Figure 8g). Those bowl-like micelles were particularly informative (Figure 9). Indeed, they appeared to consist of a base of ca. 100 nm of diameter onto which a ring rested. The “base”, itself, appeared to be composed of merged spheres. As pointed out with yellow arrows in Figure 9a,c,e, this base could be perforated, suggesting that the overall structure might be the precursor of the wedding rings.

## 4. Discussion

The observation of bowl-like micelles (even somewhat ill-defined) and “wedding rings” when varying the overall solvent polarity was quite unique since the temperature used for these experiments (32 °C), was equivalent of the Tg of a polystyrene with 13 units [47], far below the Tg of a high molecular weight PS (100 °C).

To have a better understanding of what could cause the formation of these less common structures (for high Tg polymers), it is important to recall that a key aspect of PISA is the presence of a large amount of unimer in the reaction medium [48]. Indeed, as the BCP self-assembles, the micelle cores can be swollen by the monomer, strongly affecting their viscosity as well as their polymerization kinetics [49]. Monomer partitioning thus plays a crucial role by swelling and softening the core, favoring morphological transitions.

**Figure 10:**
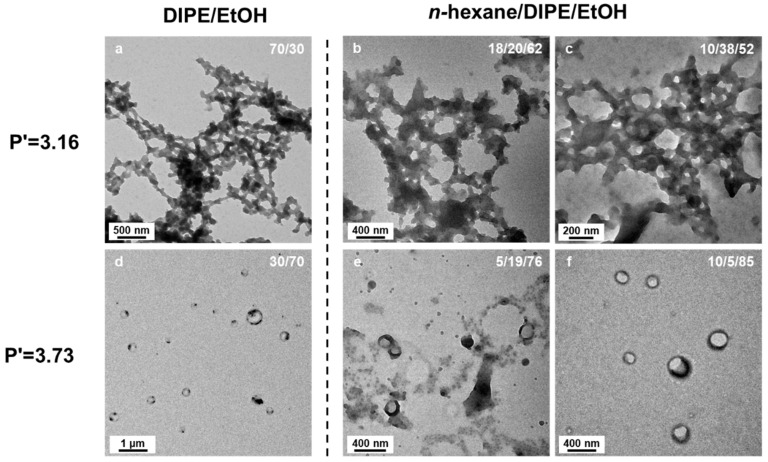
TEM images of P*t*BA_42_-*b*-PS_m_ micelle morphologies in binary and ternary solvent systems. The samples were prepared at a polarity P’ = 3.16 using solvent mixtures of (**a**) DIPE/EtOH (70/30 *v*/*v*%), (**b**) *n*-hexane/DIPE/EtOH (18/20/62, *v*/*v*/*v*%), (**c**) *n*-hexane/DIPE/EtOH (10/38/52, *v*/*v*/*v*%) and at a polarity of P’ = 3.73 in solvent mixtures of (**d**) DIPE/EtOH (30/70 *v*/*v*%), (**e**) *n*-hexane/DIPE/EtOH (5/19/76, *v*/*v*/*v*%) and (**f**) *n*-hexane/DIPE/EtOH (10/5/85, *v*/*v*/*v*%) The experiments were performed at 32 °C, with 25 wt% solids content, an incident lght power of 5.5 W, at 405 nm wavelength for 15 h).

The behavior of polymers dissolved in solvent mixtures can be extremely complex [50], with numerous possible factors that can affect the stability of micelles [51,52] and the partitioning of the monomer between the micelle core and the solvent. We were thus concerned that the variations in morphology of the nanostructures formed as a function of the solvent ratio would be governed by different phenomena, such as preferential adsorption of one of the solvents, rather than simply a change in solvent polarity. We thus decided to prepare three different solvent mixtures with equivalent polarity index (Figure 10), P’ = 3.16, one binary solvent mixture consisting of DIPE/EtOH (70/30 *v*/*v*%) and two *n*-hexane/DIPE/EtOH ternary mixtures, namely *n*-hexane/DIPE/EtOH (18/20/32 *v*/*v*/*v*%) and (10/38/52 *v*/*v*/*v*%) that would also have a polarity index P’ = 3.16 (see details of the calculation of the polarity indices in Supporting Information). Here, our rationale was that such a complex (ternary) solvent system would certainly affect the final morphologies of the micelles if solvent polarity was the main driving force of monomer partitioning and morphological transitions.

As shown in Figure 10, the structures obtained in those three different solvent systems after 15 h of irradiation at 32 °C using P*t*BA_42_ as macro-CTA and with 25 wt% of solids content were remarkably similar. The meso-structures formed consisted of worm-like networks connected by large hubs. However, because the worm-like network is a rather common morphology, we further confirmed the prevalence of polarity effects on our PISA formulations by studying two more solvent mixtures, aiming at a polarity index of P’ = 3.73: DIPE/EtOH (30/70 *v*/*v*%), *n*-hexane/DIPE/EtOH (5/19/76 *v*/*v*/*v*%) and *n*-hexane/DIPE/EtOH (10/5/85 *v*/*v*/*v*%). At this polarity, in these three different solvent mixtures, the micelle morphologies were, once again, essentially similar, looking like rings or broken vesicles of ca. 300 nm diameter. These results strongly supported the hypothesis that solvent polarity is most likely the main driving force favoring polymerization and morphological transitions.

**Figure 11:**
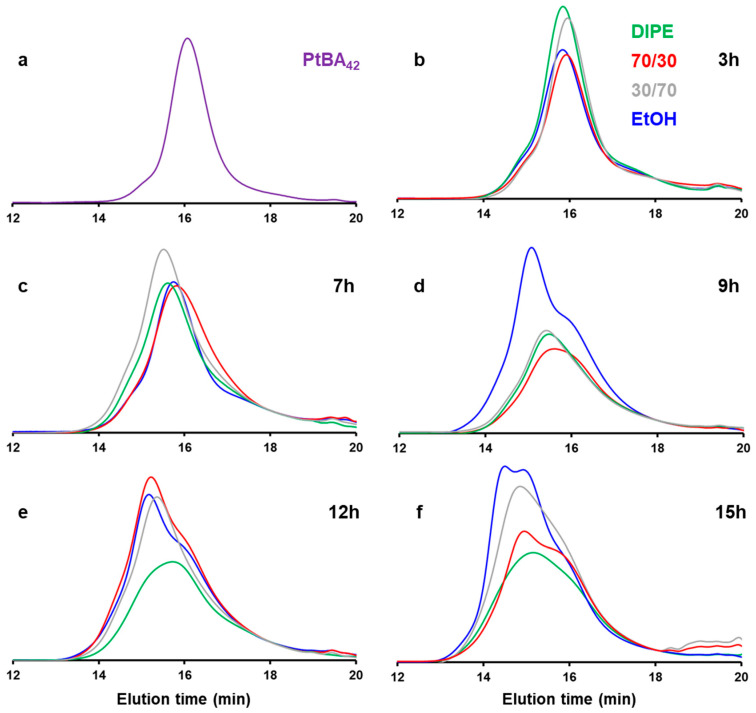
GPC traces of (**a**) P*t*BA_42_, and DIPE (P’ = 2.40) (green), DIPE/EtOH (70/30 *v*/*v*%) (P’ = 2.97) (red), DIPE/EtOH (30/70 *v*/*v*%) (P’ = 3.73) (grey) and EtOH (P’ = 4.30) (blue) after (**b**) 3 h, (**c**) 7 h, (**d**) 9 h, (**e**) 12 h and (**f**) 15 h of reaction time. Note that the RI values were normalized using an elution time of 18 min as a reference.

**Figure 12:**
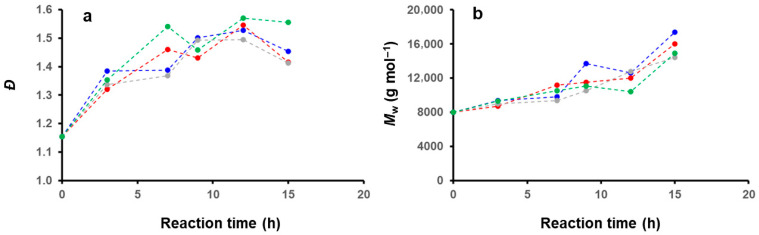
Plots of the (**a**) molecular weight distributions (**b**) weight average molecular weights and as a function of reaction time for PISA reactions performed in DIPE (green), DIPE/EtOH (70/30 *v*/*v*%) (red), DIPE/EtOH (30/70 *v*/*v*%) (grey) and EtOH (blue).

We then further investigated the effect of the solvent medium on the PISA by performing kinetics experiments by GPC (Figure 11). For this purpose, we selected four different mixtures (DIPE/EtOH: 100/0, 70/30, 30/70, 0/100 *v*/*v*%), and performed five reactions in parallel that we stopped at different reaction times: 3, 7, 9, 12 and 15 h. The main characteristics of the polymer obtained (*M*_w_, Ɖ) are shown in Figure 12. Interestingly, despite the morphological differences observed for these samples after 15 h of reaction (cf. Figure 1, Figure 3, Figure 5 and Figure 6), the GPC traces remained relatively similar, and the weight average molecular weights obtained even after 15 h of reaction time were relatively low (ca. 16 kDa on average), indicating that the reactions were particularly slow throughout the PISA experiments.

As we wanted to have a complete picture of our PISA experiments, we chose to follow the same cleaning procedure as that used to clean the P*t*BA initiator: after evaporation of the solvent, we dissolved the solid in a minimum amount of acetone and added a mixture of methanol/water (50/50 *v*/*v*%) to precipitate the polymer. After centrifugation, we removed the supernatant and reperformed the cycle of dissolution–precipitation–centrifugation twice more.

Since the amount of polymer injected was different from one sample to another, we plotted normalized GPC traces. In the present case, we were not expecting to see polymers with molecular weights lower than that of the macro-P*t*BA. We thus normalized the refractive index (RI) values using an elution time of 18 min as a reference. We believe that this representation better emphasizes the evolution of the molecular weights of the samples as a function of reaction time.

The first observation in Figure 11 is the presence of what appears to be unreacted mP*t*BA_42_. Interestingly, with the exception of the PISA performed in ethanol, the GPC traces of the three other solvent systems investigated did not present obvious differences. They all showed bimodal distributions with a noticeable amount of mP*t*BA_42_, while the second peak, located at shorter elution time, became more prominent with reaction time, suggesting that mPtBA_42_ was slowly being consumed. This observation was also confirmed by the overall trend of the plot of the molecular weight distributions versus time which appeared to plateau, after an initial increase due the partial polymerization of mP*t*BA. In ethanol, the reaction appeared to be slightly faster with the presence of a third population at a much shorter reaction time, equivalent to a molecular weight of 23 kDa.

Presence of different populations of block copolymers (including most likely mP*t*BA) in the reaction medium prevented us from performing a quantitative study by ^1^H NMR, and only semiquantitative values of the degree of polymerizations of the PS blocks were accessible (Table S1). These data were used to approximate the yield of the reactions, assuming that mP*t*BA was fully recovered by our sample cleaning protocol, which was, unfortunately, not always the case. Since for all the experiments performed, we used 200 equivalents of styrene, the reaction yield (%) would simply be given by DP_styrene_/2.

As shown in Figure 12b, the weight average molecular weights of the different samples remained very similar, suggesting that the solvent system had only little impact on the polymerization kinetic of P*t*BA_42_-*b*-PS. This observation is particularly interesting, and points toward a key aspect of the PISA of core-forming polymers with high Tg. As one would expect, and as confirmed by our kinetic study, the core of the micelle is mostly in a frozen state, strongly slowing the main process involved in the morphological transitions observed during PISA of core forming block with low Tg, i.e., polymerization, chain exchange (between micelles) and micelle fusion [53].

Although thermodynamic models may not fully apply to micelles with kinetically trapped cores, they can provide partial insight into the role of solvent polarity on micelle morphologies. In the following paragraphs, we are proposing a tentative explanation of the morphological transitions for PISA performed with mP*t*BA_42_.

For micellar systems at equilibrium [54,55], the total free energy per chain could be written as: *F*_chain_ = *F*_interface_ + *F*_core_ + *F*_corona_. However, since the polymerization of styrene did not appear to be strongly affected by the solvent polarity, we could discard the free energy of the micelle core which is proportional to the molecular weight of the core forming block [54]:

*F*_chain_ ≈ *F*_interface_ + *F*_corona_.

Comparison between the Hansen solubility parameters of PS, styrene, diisopropyl ether and ethanol (Table S2) indicates that styrene is less miscible with ethanol than with diisopropyl ether, but can solubilize PS. One should thus expect to observe preferential adsorption of styrene on the micelle cores, leading to an enrichment of styrene at the interface between the PS core of the micelle and the solvent. As the solvent polarity was increased, preferential adsorption of styrene was most likely enhanced. However, as a first approximation, we consider that the interfacial free energy would be relatively unaffected by the change in solvent polarity due to the saturation of styrene at the interface between the core and the solvent system for all solvent mixtures.

Therefore, we hypothesize that the main factor influencing the micelle morphology is the interactions between the corona and solvent. This is particularly true when mixtures of protic polar solvent and apolar solvents are considered. Manners, O’reilly and coworkers [56], for example, showed that the crystallization-driven self-assembly of poly(*L*-lactide)-*block*-poly(*N* isopropylacrylamide) (PLLA-*b*-PNIPAM) block copolymer could be improved by using trifluoroethanol (TFE) as cosolvent. The authors explained this effect by an enhancement of the solubility of the corona as TFE disrupted the PLLA intermolecular hydrogen bonding.

In the present system, for small amounts of ethanol (5 v%), hydrogen bonding is mainly local and would mostly be intramolecular, increasing the local rigidity of P*t*BA. At that stage, the corona chains would be more stretched, facilitating the formation of lamellar/vesicular structures with a larger radius (Figure 1b). As the polarity of the solvent mixture was increased, the micelle core became more prone to fusion due the enhanced corona stretching, coupled with the presence of styrene at the core/solvent interface and in the core. As the high Tg of PS does not permit a complete merging of the different cores, ill-defined worm-like structures were obtained (Figure 1c, Figure 2 and Figure 3a,b). Further increased in the amount of ethanol (above 30 v%, equivalent to 50 mol%) led to the formation of intermolecular hydrogen bonding, decreasing the corona stretching and thus favoring the formation of spherical structures. In addition, intermolecular hydrogen bonding would promote interpenetration between corona from different spherical micelles and the formation of necklaces, as observed when 40 v% of ethanol was used (Figure 3c, for example). As shown in Figure 3d and Figure 4a,b, as the corona of different micelles merged, they promoted the fusion of the micelle cores leading to bowl-like structures with a flattened, lamellar, bottom section. As the volume percentage of ethanol was increased to 90%, large broken vesicular structures (kippah) could be observed (Figure 5c,f). Finally, as the solvent polarity became maximal (100 v% of ethanol), in the absence of the apolar DIPE, spherical micelles were observed again with the presence of necklace-like structures most likely induced by persistent inter-coronal hydrogen bonding.

## 5. Conclusions

In summary, we have shown that using solvent mixtures with a broad range of polarities enables the preparation of meso-structures with complex morphologies using mP*t*BA to polymerize styrene in alcoholic media. This result was rather remarkable since the morphologies were obtained near room temperature, far from the expected glass transition of high molecular weight PS (100 °C). More specifically, through the systematic modulation of the polarity of the reaction medium, we observed a morphological transition of micellar architectures from spheres to bowl-like micelles, with intermediate states including necklace-like micelles, three-dimensionally interconnected network structures and wedding rings, further increasing the number of accessible morphologies obtained for PISA with high-Tg insoluble polymers. Previously, only spheres, short worm-like and aggregated spheres had been reported. We attribute this phenomenon to both the interactions between the solvent and the corona, and the swelling of the micellar core. Presence of styrene inside the core facilitated the morphological transitions induced by corona/solvent interactions. Therefore, visible-light-initiated RAFT-PISA performed in mixed solvent systems offers a convenient approach to access highly complex structures under mild conditions.

## Data Availability

The original contributions presented in this study are included in the article/Supplementary Material. Further inquiries can be directed to the corresponding author.

## Supplementary Materials

The following supporting information can be downloaded at: https://www.mdpi.com/article/10.3390/polym18020165/s1. Reference [57] is cited in the supplementary materials. The supplementary materials include representative ^1^H NMR spectra and GPC traces of PtBA macromonomers and P*t*BA-*b*-PS block copolymers syntheized by PISA, Tables slisting the degrees of polymerization, molecular weights and yield percentages of all the polymers synthesized as evaluated by ^1^H NMR and GPC. As well as tables of the Hansen solubility parameters and the Rohrschneider polarity parameters for the different solvents and solvent mixtures used.
