# Supplementary material for: Effect of Solvent Polarity on the Photo-Induced Polymerization-Induced Self-Assembly of Poly(tert-butyl acrylate)-block-Polystyrene near Room Temperature
Source: Polymers (Basel). 2026 Jan 7;18(2):165. doi: 10.3390/polym18020165 (PMC12845964; doi:10.3390/polym18020165)
Supplement: Supplementary file 1 [file polymers-18-00165-s001.zip › polymers-4034084-supplementary.pdf]

SUPPORTING INFORMATION

Effect of solvent polarity on the photo-induced polymerization-induced self-assembly of poly(*tert*-butyl acrylate)-*block*-polystyrene near room temperature

Tianyi Zhou, Jiawei Song and Gerald Guerin*

Shanghai Key Laboratory of Advanced Polymeric Materials, Key Laboratory for Ultrafine Materials of Ministry of Education, School of Materials Science and Engineering, East China University of Science and Technology, Shanghai 200237, China

*Corresponding author: gguerin@ecust.edu.cn

Experimental Section

Materials

Tert-butyl acrylate (tBA, Aladdin) was purified by distillation at 55 °C before being stored at -30 °C. Dodecanethiol (Boer, 99%), potassium phosphate (Nicom, 99%), carbon disulfide (Boer, AR), 4-(chloromethyl)benzyl alcohol (Aladdin, 99%), chloroform (General-reagent, AR), methanol (Adamas-beta, AR), diisopropyl ether (General-reagent, AR), ethanol (Adamas-beta, AR), acetone (General-reagent, AR), *n*-hexane (General-reagent, AR), tetrahydrofuran (THF) (General-reagent, AR), NaCl (Titan, 99%) were used as received. 2,2'-Azobis-(2-methylbutyronitrile) (AMBN, Aladdin) was recrystallized from ethanol before storage under refrigeration at 4 °C. Dodecyl 4-(hydroxymethyl) benzyl carbonotrithioate chain transform agent (CTA-1) was synthesized according to the O'Reilly and coworkers' literature [1]. Sodium phenyl-2,4,6-trimethylbenzoylphosphinate (SPTP) was synthesized according to Du et al. [2].

Characterization

Proton nuclear magnetic resonance spectroscopy (^{1}H NMR). Nuclear magnetic resonance (NMR) spectra were recorded in $CDCl_3$ using a Bruker Avance III HD 400 MHz NMR spectrometer at a temperature of 25 °C. Tetramethylsilane (TMS) was used as an internal standard, and the concentration of the test samples was 10-20 mg/mL.

Gel permeation chromatography (GPC). Molecular weight and polydispersity of block copolymers were measured by GPC using a Waters 1515 GPC instrument with tetrahydrofuran (THF) as the mobile phase and Waters styragel HR1 and HR4 chromatography columns at 35 °C. THF flow rate was 1.0 mL/min, and the concentration of product was 1 mg mL^{-1}. Linear polystyrene polymers with narrow molecular weight distributions were used as standards for the calibration of the instrument.

Transmission electron microscopy (TEM). The obtained dispersions were diluted 150-fold with the corresponding solvent mixture. A drop of the solution was placed on a copper grid for 30 seconds and then blotted with a filter paper to remove excess solution. TEM observations were carried out on a JEM1400 (JEOL, Japan) instrument operated at 100 kV.

Synthesis of dodecyl 4-(hydroxymethyl) benzyl carbonotrithioate CTA (CTA-1)

Dodecanethiol (1.96 mL, 8.20 mmol,1eq.) was dissolved in acetone (19.0 ml),

potassium phosphate (1.90 g, 9.18 mmol, 1.1 eq.), and carbon disulfide (2.03 g, 26.59 mmol,3.1 eq.) were added and the solution mixture was stirred at room temperature for 2 hours. Subsequently, 4-(chloromethyl)benzyl alcohol (1 g, 6.38 mmol, 1 eq.) was added to the yellow solution, and the reaction was left for 72 hours. Afterwards, the solvent was evaporated and the remaining solid was dissolved in dichloromethane. The organic layers were then washed with 0.1 M HCl (100 mL) once, DI water (100 mL) three times, and then a saturated NaCl solution (100 mL) once. The organic phase was dried over magnesium sulfate, filtered, and concentrated in a vacuum. Subsequently, the product was recrystallized twice in hexane (10 mL). The final product was dried at room temperature in a vacuum oven overnight. The mass of the final product was 2.5g, and the conversion was 81%. The ^{1}H NMR spectrum is shown in Fig. S2.

Synthesis of P*t*BA$_n$ by RAFT polymerization

The poly(*tert*-butyl acrylate) (P*t*BA) macromonomers were all synthesized by RAFT polymerization following a similar recipe, varying solely the ratio between *t*BA and CTA1. As an example, we give below the recipe for the synthesis of mP*t*BA$_{79}$ along with the ^{1}H NMR spectrum (Figure S3) and the GPC trace (Figure S4). All the data are summarized in Table S3.

Synthesis of P*t*BA$_{79}$ by RAFT polymerization

Poly(*tert*-butyl acrylate) (P*t*BA) was prepared using CTA-1 as the chain transfer agent. CTA-1 (63.48 mg, 0.16 mmol.1 eq.), *tert*-butyl acrylate (*t*BA) (1.84 g, 14.36 mmol, 90 eq.), and AMBN (3.07 mg, 0.016 mmol, 0.1 eq.) form a stock solution in chloroform (2.08 mL) were introduced in a 25 mL round bottom flask equipped with a magnetic stirrer. The solution was done "freeze-pump-thaw" three times to remove oxygen gas completely. After being degassed under argon gas for 2 mins, the solution was immersed in a thermostatic oil bath at 70 °C for 6 h. The polymerization was then quenched by immersion in a nitrogen liquid bath and exposure to air. The polymer was precipitated from a mixture of methanol and water three times. The precipitated product was then dried under vacuum overnight and recovered as a yellow powder with a mass of 1.4539 g, a conversion of 79 %. The polymer was analyzed by THF GPC with PDI = 1.13 as determined by reference to polystyrene standards. Analysis of the ^{1}H NMR performed in deuterated chloroform ($CDCl_3$) gave a degree of polymerization DP = 79. The precipitated product was then dried under vacuum overnight. The ^{1}H NMR spectrum in shown in Figure S3 and the GPC trace in Figure S4.

PISA of P*t*BA-*b*-PS

Similarly, PISA experiments were performed following the same protocol, varying the ratio of DIPE/EtOH and/or the length of the macromonomer used. We thus give below the general recipe used with P*t*BA_{79} as macromonomer.

Synthesis of P*t*BA_{79}-*b*-PS

In a typical experiment for the synthesis of P*t*BA-*b*-PS, P*t*BA_{79} (100 mg, 0.0095 mmol, 1eq.), sodium phenyl-2,4,6-trimethylbenzoylphosphinate (SPTP) (0.66 mg, 0.0019 mmol, 0.2 eq.), and styrene (197.9 mg, 1.90 mmol, 200 eq.) were weighed into a 25 mL schlenk tube with a magnetic stirrer. The solvent mixture is prepared by mixing diisopropyl ether and ethanol in varying volume ratios (3:7, 5:5, 7:3, and 9:1 v/v). A certain amount of mixed solvent (0.64 mL, 25 wt% solid content) was added into the tube, bubbled with argon gas for 6 mins, and then irradiated by the visible light LED photo reactor (405 nm, 5.5 W) in the parallel light instrument (AL1) at 32 °C for 15 h. The polymerization was then quenched by exposure to air. The polymer was precipitated from a mixture of methanol and water three times. The ^{1}H NMR spectrum obtained from the PISA of mP*t*BA_{79} in diisopropyl ether/ethanol, 30:70, v/v reacted for 15 hr is shown in Figure S5

Visible light-initiated RAFT-PISA synthesis of P*t*BA-*b*-PS in *n*-hexane/diisopropyl ether/ethanol ternary solvent system (P' = 3.16)

In a typical experiment for the synthesis of P*t*BA-*b*-PS, P*t*BA_{42} (50 mg, 0.0086 mmol, 1 eq.), sodium phenyl-2,4,6-trimethylbenzoylphosphinate (SPTP) (0.60 mg, 0.0017 mmol, 0.2 eq.), and styrene (180.1 mg, 1.73 mmol, 200 eq.) were weighed into a 25 mL schlenk tube with a magnetic stirrer. The solvent mixture is prepared by mixing hexane, diisopropyl ether, and ethanol in varying volume ratios (18:20:62 or 10:38:52 v/v). A certain amount of mixed solvent (0.65 mL,25 wt% solid content) was added into the tube, bubbled with argon gas for 6 mins, and then irradiated by the visible light LED photo reactor (405 nm, 5.5 W) in the parallel light instrument (AL1) at 32°C for 15 h. The polymerization was then quenched by exposure to air. The polymer was precipitated from a mixture of methanol and water three times.

Visible light-initiated RAFT-PISA synthesis of P*t*BA-*b*-PS in an *n*-hexane/diisopropyl ether/ethanol ternary solvent system (P' =3.73)

In a typical experiment for the synthesis of P*t*BA-*b*-PS, P*t*BA$_{42}$ (50 mg, 0.0086 mmol, 1 eq.), sodium phenyl-2,4,6-trimethylbenzoylphosphinate (SPTP) (0.60 mg, 0.0017 mmol, 0.2 eq.), and styrene (180.1 mg, 1.73 mmol, 200 eq.) were weighed into a 25 mL schlenk tube with a magnetic stirrer. The solvent mixture is prepared by mixing hexane, diisopropyl ether, and ethanol in varying volume ratios (5:19:76 or 10:5:85 v/v). A certain amount of mixed solvent (0.65 mL,25 wt% solid content) was added into the tube, bubbled with argon gas for 6 mins, and then irradiated by the visible light LED photo reactor (405 nm, 5.5 W) in the parallel light instrument (AL1) at 32°C for 15 h. The polymerization was then quenched by exposure to air. The polymer was precipitated from a mixture of methanol and water three times.

Construction of mixed solvent systems of increasing polarity

In selective organic solvent systems, variations in solvent polarity significantly influence the solvation behavior of polymer block segments, thereby inducing and driving the self-assembly of polymers to form various micellar structures, including spherical, worm-like, vesicle, and other distinctive morphologies. Therefore, solvent selection must be performed before commencing the preparation.

We screened common laboratory solvents based on solubility tests and solvent polarity data (Tables S2 and S4).

According to the solubility data from Table S4, we chose diisopropyl ether (Polarity index, P' = 2.4) and ethanol (P' = 4.3) to compose a mixed solvent system (P' = 2.4 - 4.3), and simulated the gradient change in polarity gradient of mixed solvent from 2.4 - 4.3 with a 10% difference in volume ratio (Table S5).

Supporting Figures

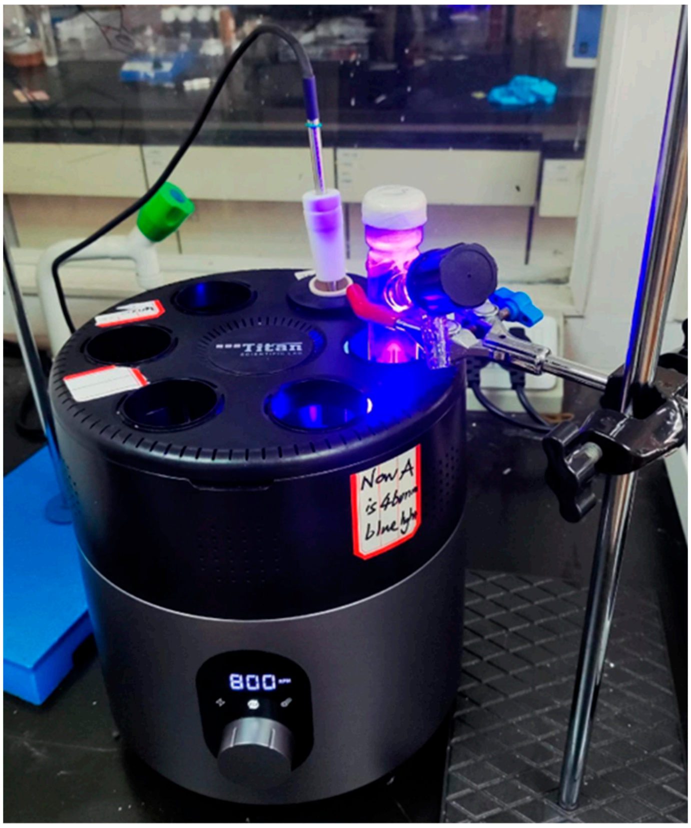

Figure S1: Experimental setup for photoinitiated RAFT-PISA (six well reactor with irradiation light wavelength and power adjustable separately).

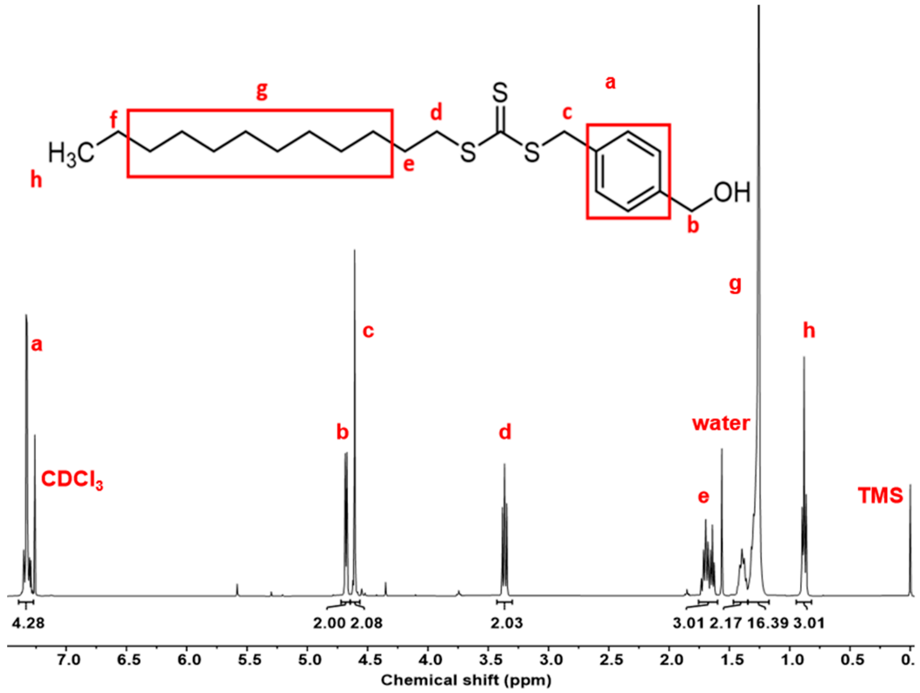

Figure S2. ^{1}H NMR spectrum of dodecyl 4-(hydroxymethyl) benzyl carbonotrithioate (600 MHz, $CDCl_3$).

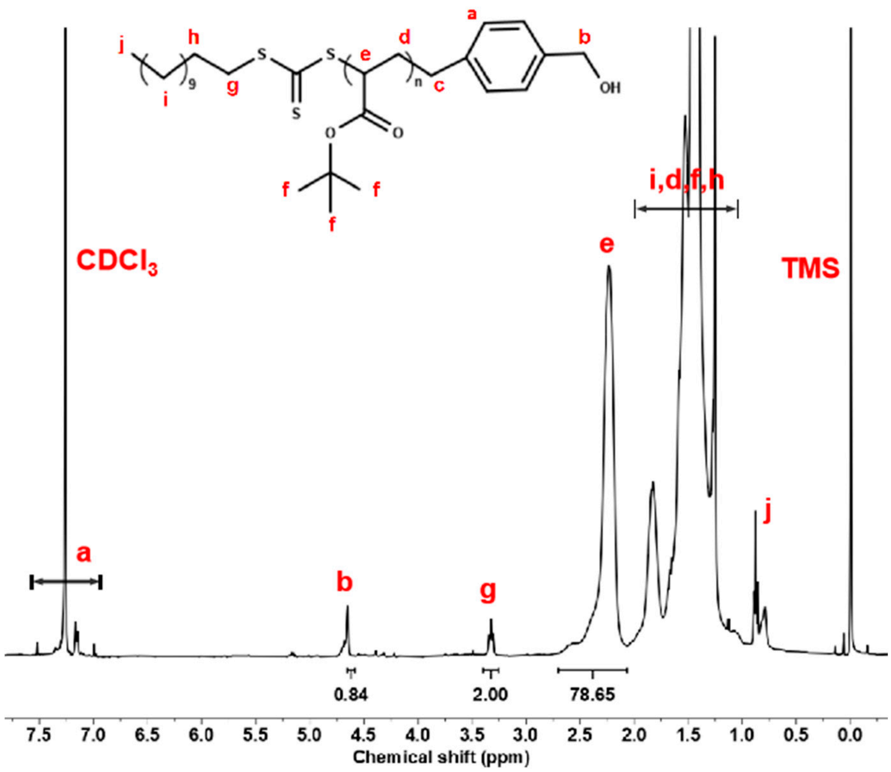

Figure S3: ^{1}H NMR of mP*t*BA$_{79}$ prepared by thermal RAFT polymerization. (400 MHz, in $CDCl_3$)

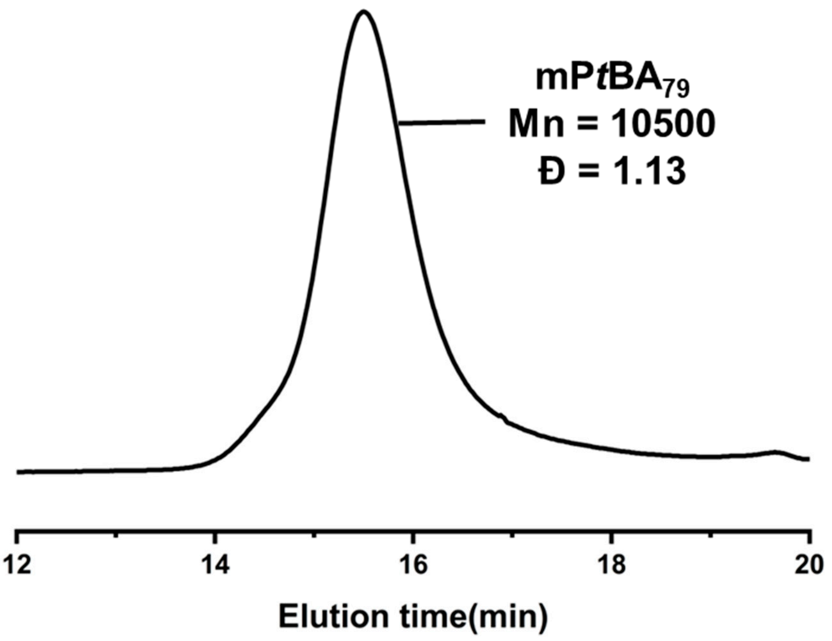

Figure S4. GPC trace of mP*t*BA$_{79}$

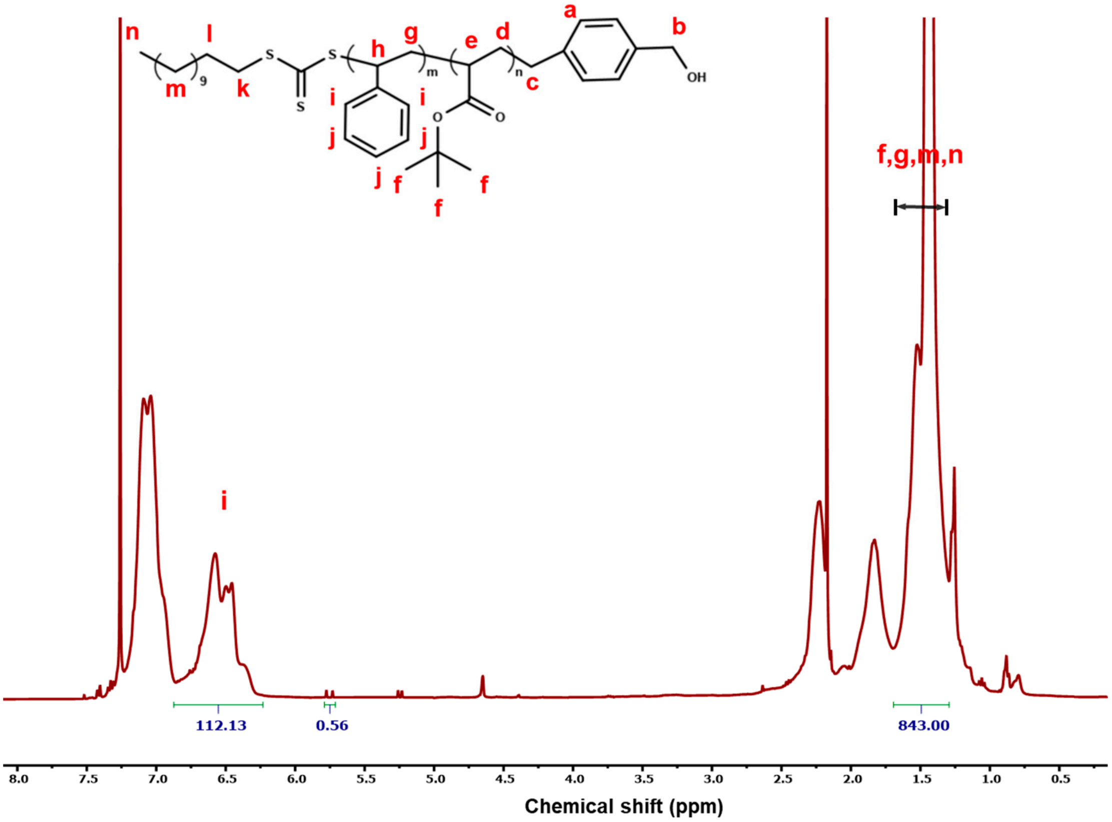

Figure S5. ^{1}H NMR spectrum (400 MHz, in $CDCl_3$) of P*t*BA$_{79}$-*b*-PS$_{56}$ diblock copolymer synthesized via visible light-induced RAFT-PISA in diisopropyl ether/ethanol (30:70, v/v%). The experiment was performed by irradiating mP*t*BA$_{79}$ for 15 h at a wavelength of 405 nm. The photoreactor power was set to 5.5 W. In some occasions, the acetone peak is present at 2.17 ppm and overlaps with the signal from the (-C*H*-) of P*t*BA. To evaluate the DP of PS in these conditions, we integrated the region between 1.25 and 1.7 ppm. This region includes the protons of the $(CH_3)_3$ tert-butyl groups of P*t*BA, the 20 protons of the CTA $(CH_2)_{10}$, and the (CH_2) protons of the PS backbone. The DP of polystyrene is then calculated by comparing the sum between the number of proton i (from 6.25 to 6.85 ppm) of the styrene groups plus the 20 protons from the CTA and finally the protons of the 79 tert-butyl groups. In the present case, that gives: 79*9+20+112 = 843. The data have to be self-consistent and thus the integration needs to be adjusted via 3 or 4 rounds to obtain the correct value.

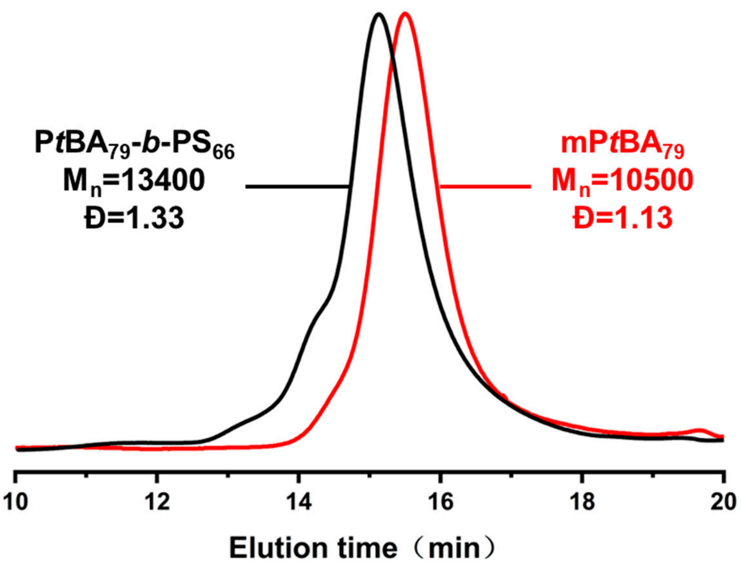

Figure S6: GPC traces of samples before and after reaction. (The red curve is the GPC curve of mP*t*BA$_{79}$ block, and the black curve is the GPC curve of P*t*BA$_{79}$-*b*-PS$_{66}$ block copolymer)

Supporting Tables

Table S1. Summary of 1H NMR data of some of the polymers synthesized by photo-induced PISA. The samples were irradiated at an intensity of 5.5 W.

Sample	Solvent Ratio (DIPE:EtOH)	DP[a] mP*t*BA	Reaction Time (h)	DP[a] PS	Yield[a] (%)	$M_{n,GPC}$ (g mol^{-1})	$M_{w,GPC}$ (g mol^{-1})	Đ
P*t*BA$_{24}$-*b*-PS$_{23}$	30:70	24	15	23	11.5	6800	12200	1.79
P*t*BA$_{36}$-*b*-PS$_{22}$	30:70	36	15	22	11	6000	15200	1.13
P*t*BA$_{42}$-*b*-PS$_{108}$	30:70	42	15	108	54	10200	14500	1.41
P*t*BA$_{56}$-*b*-PS$_{18}$	30:70	56	15	18	9	9100	12000	1.29
P*t*BA$_{79}$-*b*-PS$_{56}$	30:70	79	15	55	27.5	11200	18500	1.66
P*t*BA$_{42}$-*b*-PS$_{58}$	100:0	42	15	58	29	9600	15000	1.60
P*t*BA$_{42}$-*b*-PS$_{62}$	70:30	42	15	62	31	11300	16100	1.41
P*t*BA$_{42}$-*b*-PS$_{52}$	50:50	42	15	52	26	13400	24800	1.85
P*t*BA$_{42}$-*b*-PS$_{108}$	30:70	42	15	108	54	10200	14400	1.41
P*t*BA$_{42}$-*b*-PS$_{87}$	0:100	42	15	87	43.5	12000	17400	1.45
P*t*BA$_{42}$-*b*-PS$_{8}$	100:0	42	3	8	4	6900	9300	1.35
P*t*BA$_{42}$-*b*-PS$_{19}$	100:0	42	7	19	9.5	6900	10500	1.54
P*t*BA$_{42}$-*b*-PS$_{20}$	100:0	42	9	20	10	7600	11800	1.46
P*t*BA$_{42}$-*b*-PS$_{19}$	100:0	42	12	19	9.5	6000	10400	1.75
P*t*BA$_{42}$-*b*-PS$_{58}$	100:0	42	15	58	29	9600	15000	1.60
P*t*BA$_{42}$-*b*-PS$_{16}$	30:70	42	3	16	8	6800	9000	1.34
P*t*BA$_{42}$-*b*-PS$_{24}$	30:70	42	5	24	12	7300	10600	1.45
P*t*BA$_{42}$-*b*-PS$_{16}$	30:70	42	7	16	8	6800	9300	1.39
P*t*BA$_{42}$-*b*-PS	30:70	42	9	-	-	7100	10500	1.49
P*t*BA$_{42}$-*b*-PS$_{15}$	30:70	42	11	15	7.5	8500	13100	1.54
P*t*BA$_{42}$-*b*-PS$_{25}$	30:70	42	12	25	12.5	8500	12800	1.49
P*t*BA$_{42}$-*b*-PS$_{27}$	30:70	42	13	27	13.5	7700	11900	1.54
P*t*BA$_{42}$-*b*-PS$_{27}$	30:70	42	14	27	13.5	9200	14400	1.56
P*t*BA$_{42}$-*b*-PS$_{108}$	30:70	42	15	108	54	10200	14400	1.41
P*t*BA$_{42}$-*b*-PS$_{10}$	70:30	42	3	10	5	6600	8700	1.32
P*t*BA$_{42}$-*b*-PS$_{12}$	70:30	42	7	12	6	8100	11500	1.43
P*t*BA$_{42}$-*b*-PS$_{36}$	70:30	42	9	36	18	7600	11200	1.46
P*t*BA$_{42}$-*b*-PS$_{33}$	70:30	42	12	33	16.5	7800	12000	1.55
P*t*BA$_{42}$-*b*-PS$_{87}$	70:30	42	15	87	43.5	11300	16100	1.41
P*t*BA$_{42}$-*b*-PS$_{8}$	0:100	42	3	8	4	6800	9400	1.39
P*t*BA$_{42}$-*b*-PS$_{12}$	0:100	42	7	12	6	7100	9800	1.39
P*t*BA$_{42}$-*b*-PS$_{14}$	0:100	42	9	14	7	9100	13700	1.50
P*t*BA$_{42}$-*b*-PS$_{21}$	0:100	42	12	20	10	8300	12600	1.53
P*t*BA$_{42}$-*b*-PS$_{72}$	0:100	42	15	72	36	12000	17400	1.45

[a] Calculated from ^{1}H NMR

Table S2. Summary of the Hansen solubility parameters of main solvents and PS

	δ_d (MPa$^{0.5}$)	δ_p(MPa$^{0.5}$)	Δ_H(MPa$^{0.5}$)	δ_t(MPa$^{0.5}$)
PS	18.7	5.9	3.5	19.9
Hexane	14.9	0	0	14.9
ethanol	15.8	8.8	19.4	26.5
diisopropyl ether	15.1	3.2	3.2	15.8
Styrene	18.6	1	4.1	19.1

δ_d, δ_p, δ_H are the dispersion, polar and hydrogen bonding components of the Hansen solubility parameters and δ_t is the value of the total Hansen solubility parameter.

Table S3. Properties of macro-CTA mP*t*BA

Sample	$[M]_0/[CTA]_0/[I]_0$	Mass Yield	DP[a]	$M_{n,NMR}$	$M_{n,GPC}$	Đ
mP*t*BA$_{24}$	30:1:0.1	75.8%	24	3100	4200	1.18
mP*t*BA$_{36}$	50:1:0.1	77.6%	36	4600	5100	1.13
mP*t*BA$_{42}$	50:1:0.1	76.6%	42	5400	5900	1.15
mP*t*BA$_{45}$	50:1:0.1	81.2%	45	6300	6300	1.18
mP*t*BA$_{56}$	70:1:0.1	82.4%	56	7200	7700	1.17
mP*t*BA$_{79}$	90:1:0.1	81.2%	79	10100	10600	1.13

[a] Determined by ^{1}H NMR

$M_{n, NMR}$: Calculated from the DP given by ^{1}H NMR. $M_n = DP*M_{monomer} + M_{CTA\ agent}$, where $M_{monomer}$ is the molecular weight of mP*t*BA;

$M_{n, GPC}$: Determined by GPC in THF using PS standards.

Table S4. Solubilization of mP*t*BA and PS in different solvents

Solvent	Rohrschneider Polarity index (P')[3,4]	mP*t*BA	PS
n-hexane	0.1	Poorly soluble	Insoluble
Diisopropyl ether (DIPE)	2.4	Soluble	Insoluble
Dichloromethane (DCM)	3.1	Soluble	Soluble
Isopropanol (IPA)	3.9	Soluble	Insoluble
Tetrahydrofuran (THF)	4.0	Soluble	Soluble
Chloroform	4.1	Soluble	Soluble
Ethanol	4.3	Soluble	Insoluble
Methanol	5.1	Soluble	Insoluble
Acetonitrile (ACN)	5.8	Soluble	Insoluble
Water	10	Insoluble	Insoluble

P.S.: All solubility tests were conducted at room temperature (25 ± 1°C) with polymers of specific degrees of polymerization: mP*t*BA (DP = 176) and PS (DP = 160). The Rohrschneider polarity parameters (P') for solvents in Table S4 were sourced from Shodex Co., Ltd. The solubility behavior of mP*t*BA and PS was experimentally determined through standardized dissolution tests.

Table S5. Polarity values of the mixed solvent system

Volume ratio v1/(v1+v2)	Polarity index of mixed solvents(P')
0	2.40
0.05	2.49
0.10	2.59
0.20	2.78
0.30	2.97
0.40	3.16
0.50	3.35
0.60	3.54
0.70	3.73
0.80	3.92
0.90	4.11
0.95	4.21
1.00	4.30

The volume fraction of ethanol was defined as x_1, and that of diisopropyl ether as x_2.

a. The polarity index (P') of the mixed solvent system is calculated by the volume fractio*n*-weighted average of the polarity indices of the pure solvents.

References

1. Petzetakis, N.; Dove, A.P.; O'Reilly, R.K. Cylindrical Micelles from the Living Crystallization-Driven Self-Assembly of Poly(Lactide)-Containing Block Copolymers. *Chem. Sci.* **2011**, *2*, 955, doi:10.1039/c0sc00596g.
2. Du, Y.; Jia, S.; Chen, Y.; Zhang, L.; Tan, J. Type I Photoinitiator-Functionalized Block Copolymer Nanoparticles Prepared by RAFT-Mediated Polymerization-Induced Self-Assembly. *ACS Macro Lett.* **2021**, *10*, 297–306, doi:10.1021/acsmacrolett.1c00014.
3. Rohrschneider, L. Chromatographic Characterization of Liquid Phases and Solutes for Column Selection and Identification. *Journal of Chromatographic Science* **1973**, *11*, 160–166, doi:10.1093/chromsci/11.3.160.
4. Snyder, L.R. Classification of the Solvent Properties of Common Liquids. *Journal of Chromatography A* **1974**, *92*, 223–230, doi:10.1016/S0021-9673(00)85732-5.
